# Microstructural Gradational Properties of Sn-Doped Gallium Oxide Heteroepitaxial Layers Grown Using Mist Chemical Vapor Deposition

**DOI:** 10.3390/ma15031050

**Published:** 2022-01-29

**Authors:** Kyoung-Ho Kim, Minh-Tan Ha, Heesoo Lee, Minho Kim, Okhyun Nam, Yun-Ji Shin, Seong-Min Jeong, Si-Young Bae

**Affiliations:** 1 Semiconductor Materials Center, Korea Institute of Ceramic Engineering and Technology, Jinju 52851, Korea; energykkh@gmail.com (K.-H.K.); shinyj@kicet.re.kr (Y.-J.S.); smjeong@kicet.re.kr (S.-M.J.); 2 School of Materials Science and Engineering, Pusan National University, Busan 46241, Korea; heesoo@pusan.ac.kr; 3 School of Materials Science and Engineering, Changwon National University, Changwon 51140, Korea; haminhtan.mse@gmail.com; 4 Convergence Center for Advanced Nano Semiconductor (CANS), Department of Nano & Semiconductor Engineering, Korea Polytechnic University, Siheung-si 15073, Korea; First_kim88@naver.com (M.K.); ohnam@kpu.ac.kr (O.N.)

**Keywords:** Gallium oxide, mist CVD, doping, epitaxy, microstructural gradation, scattering, two-step growth

## Abstract

This study examined the microstructural gradation in Sn-doped, n-type Ga_2_O_3_ epitaxial layers grown on a two-inch sapphire substrate using horizontal hot-wall mist chemical vapor deposition (mist CVD). The results revealed that, compared to a single Ga_2_O_3_ layer grown using a conventional single-step growth, the double Ga_2_O_3_ layers grown using a two-step growth process exhibited excellent thickness uniformity, surface roughness, and crystal quality. In addition, the spatial gradient of carrier concentration in the upper layer of the double layers was significantly affected by the mist flow velocity at the surface, regardless of the dopant concentration distribution of the underlying layer. Furthermore, the electrical properties of the single Ga_2_O_3_ layer could be attributed to various scattering mechanisms, whereas the carrier mobility of the double Ga_2_O_3_ layers could be attributed to Coulomb scattering owing to the heavily doped condition. It strongly suggests the two-step-grown, lightly-Sn-doped Ga_2_O_3_ layer is feasible for high power electronic devices.

## 1. Introduction

Recently, there has been a rapid increase in the demand for high electronic power devices, such as electric vehicles, industrial machines, and smart grids [1,2,3]. Next-generation power devices, which are based on Si semiconductors, have limited working voltages, frequencies, and efficiencies owing to the natural narrow bandgap of Si [4,5,6]. Therefore, ultra-wide bandgap materials, such as SiC, GaN, and Ga_2_O_3_, have emerged as promising alternatives to Si in next-generation, high-efficiency power devices. Among these materials, Ga_2_O_3_, a polymorphic crystalline material, exhibits a high bandgap in the range from 4.6 to 5.3 eV and a high breakdown field of approximately 8 MV/cm [1,7,8]. Among various Ga_2_O_3_ polytypes, the corundum-structured Ga_2_O_3_ (namely α-Ga_2_O_3_) exhibits the highest bandgap (5.3 eV) [9,10]. Moreover, owing to its corundum structure, the bandgap of α-Ga_2_O_3_ can be tuned by alloying it with α-Al_2_O_3_ or α-In_2_O_3_, which exhibit similar corundum structures [11]. In addition, owing to the unavailability of high quality, large scale, and native Ga_2_O_3_ substrates, it is essential to develop hetero-epitaxial growth methods for fabricating high-quality α-Ga_2_O_3_ crystalline films with controlled electrical properties.

To date, α-Al_2_O_3_ (sapphire substrate) is regarded as the best foreign substrate for α-Ga_2_O_3_ epitaxy, owing to their small lattice mismatch [9,11] and the large-scale commercial availability and reasonable cost of sapphire wafers. Accordingly, several methods have been employed for the hetero-epitaxial growth of Ga_2_O_3_, such as molecular beam epitaxy [12], halide vapor phase epitaxy [13], metal-organic chemical vapor deposition [14], and mist chemical vapor deposition (mist CVD) [15]. Among these methods, the mist CVD method is a green, facile, and economical approach for the epitaxial growth of phase-pure α-Ga_2_O_3_ on sapphire substrates [15,16].

Unintentionally doped α-Ga_2_O_3_ epitaxial layers exhibit semi-insulating properties with a high resistivity (>1 × 10^5^ Ωcm), owing to their low background impurity [17]. Group VI elements, such as Sn and Si, have been commonly adopted as dopants for α-Ga_2_O_3_ to achieve n-type properties. In particular, owing to the similar size of Sn and Ga at the cation sites (radius of 0.69 and 0.62 Å for Sn and Ga, respectively) [18], Sn can be easily substituted with Ga in the Ga_2_O_3_ crystal structure without a significant lattice distortion. Tin(II) chloride has attracted widespread attention for doping Ga_2_O_3_ epilayers in a mist CVD system owing to its tractability [16,17,18]. However, several scattering mechanisms (e.g., impurity, lattice (or phonon), dislocation, and surface roughness) deteriorate the electrical properties of the fabricated Sn-doped α-Ga_2_O_3_ epitaxial layers [19,20,21,22].

Moreover, the microstructural gradations, which are spatial gradients of thickness and roughness depending on the substrate position of Ga_2_O_3_ layers, can result in an under-control of the doping uniformity due to the variation in the velocity of the mist flow on the substrate in the mist CVD system [23]. Thus, in this study, we compared the various scattering components that can affect the electrical properties of two different Sn-doped Ga_2_O_3_ epitaxial layers: a single Ga_2_O_3_ layer fabricated using a conventional one-step mist CVD growth and double Ga_2_O_3_ layers fabricated using a two-step growth method.

## 2. Materials and Methods

**Figure 1:**
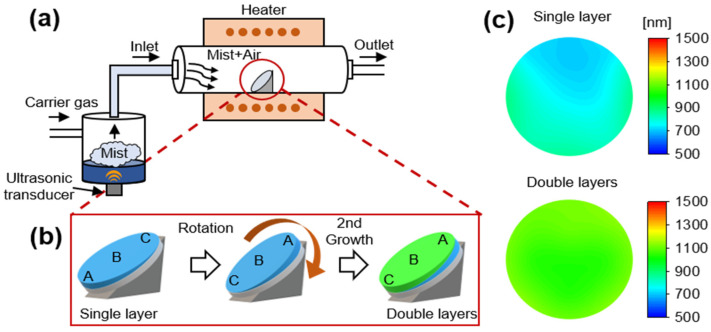
(**a**) Schematic of a horizontal hot-wall mist chemical vapor deposition (CVD). (**b**) Experimental procedure for the fabrication of the single- and double-layer growth. A (at the primary flat of the substrate), B (at the center of the substrate), and C (at opposite position from A) points indicate the orientation of the substrate in the reactor. (**c**) The thickness distributions of the single-layer and double-layer samples.

Sn-doped Ga_2_O_3_ layers were fabricated on sapphire substrates using horizontal hot-wall mist CVD, which is schematically illustrated in Figure 1a. The precursor solutions used for the preparation process were 0.05 mol/L gallium (III) acetylacetonate Ga(C_5_H_7_O_2_)_3_ 99.99% (Alfa Aesar, Haverhill, MA, USA) and 5 × 10^−7^ mol/L tin (II) chloride SnCl_2_ ≥99.995% (Sigma-Aldrich, Saint Louis, MO, USA) in acidic (pH = 1) water (Sn:Ga ratio = 0.1%). The mist was generated using a 1.7 MHz ultrasonic transducer placed beneath the solution at a temperature of 30 °C. The mist was transferred by air into a reactor at a rate of 25 L/min. The reactor was a quartz tube with a diameter and length of 10 and 100 cm, respectively, and was surrounded by a resistive furnace with a length of 40 cm and an operating temperature of 500 °C. A substrate was placed on a holder inside the quartz tube at an angle of 45°. During the deposition, the mist transfers to the substrate to feed the source materials of thin films.

According to previous studies [15,24], fabricating a Ga_2_O_3_ layer by the conventional horizontal hot-wall mist CVD exhibits a poor thickness uniformity, which resulted in a sloped single layer (Figure 1b). Hence, we developed a two-step growth process with an initial idea of improving the thickness uniformity. In the two-step growth process, first, the substrate was taken out of the reactor after the conventional growth (the first step), after which the substrate was rotated 180°, loaded back to the furnace, and deposited for the second layer (the second step) to achieve improved thickness uniformity in the double Ga_2_O_3_ layers (Figure 1b). The growth conditions and time for the first and second steps are exactly the same.

The thickness of the grown layers was measured using spectral reflectometry (F20-UV, FILMETRICS, San Diego, CA, USA). The thickness of the grown layers was verified using field-emission scanning electron microscopy (FE-SEM; JSM-7610F, JEOL, Tokyo, Japan), and the crystal structure of the grown layers was investigated using X-ray diffraction (XRD) with a Cu Kα1 radiation (λ = 1.54056 Å). An ω-scan was performed using high-resolution XRD (SMARTLAB, Rigaku, Tokyo, Japan) to examine the crystal quality. The surface roughness of the grown epilayer was investigated using atomic force microscopy (AFM; JSPM-5200, JEOL, Tokyo, Japan), and the dislocation in the grown layers was investigated using transmission electron microscopy (TEM; Themis Z, Thermo Fisher Scientific, Waltham, MA, USA). The mobility and carrier concentration of the sample were examined using Hall measurement systems at low temperatures (HMS-5000, ECOPiA, Anyang, Korea) and at room temperature (HMS-2000, ECOPiA, Anyang, Korea). The injection current of the Hall measurement was set in the range of 10–100 µA with a magnetic field of 0.52 T.

## 3. Results and Discussion

### 3.1. Microstructural Gradation of the Ga_2_O_3_ Layers

**Figure 2:**
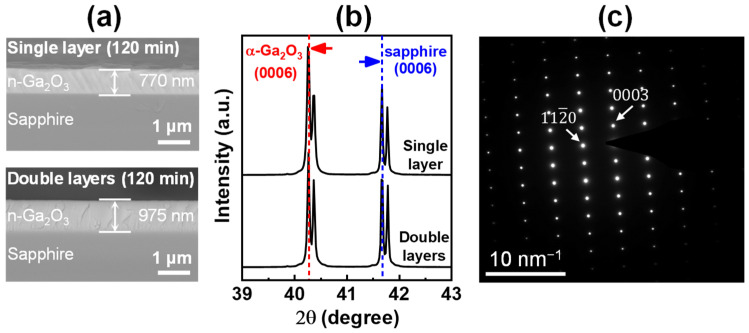
(**a**) Cross-sectional field emission scanning electron microscopy (FE-SEM) images and thickness of the single layer and double layers samples grown for 120 min. (**b**) 2θ-scan X-ray diffraction (XRD) patterns of the samples. (**c**) Electron diffraction pattern of the double layers sample.

Figure 1c shows the thickness distribution contour maps of the grown Ga_2_O_3_ layers at a growth time of 120 min. The thickness of the single Ga_2_O_3_ layer over the substrate ranged from 700 to 900 nm. As reported in previous studies [15,24], the thickness of the grown Ga_2_O_3_ layers reduces from the lower to the upper position of the substrate, owing to the increase in the velocity of the mist flow during the horizontal mist CVD. In contrast, the thickness of the double Ga_2_O_3_ layers over the substrate was in the range of 1055 ± 85 nm, indicating a high thickness uniformity. The cross-sectional SEM images of the areas around the center of the substrates (position B) of the single and double Ga_2_O_3_ layers are shown in Figure 2a. Owing to the relative smoothness of the grown layers, the actual growth thickness was consistent with the values obtained by spectral reflectometry. Hence, the double layers exhibited improved thickness uniformity. Figure 2b shows the 2θ XRD scan of the Ga_2_O_3_ layers grown on a c-plane sapphire substrate. The (0006) plane peaks of sapphire and α-Ga_2_O_3_ were simultaneously observed at 41.67 and 40.25° in the XRD patterns of the single and double layers, respectively [25]. In addition, the diffraction pattern of the double Ga_2_O_3_ layers observed along the 〈$10\bar{1}0$〉 zone axis further confirmed the corundum structure of the α-Ga_2_O_3_ crystal with TEM analysis.

**Figure 3:**
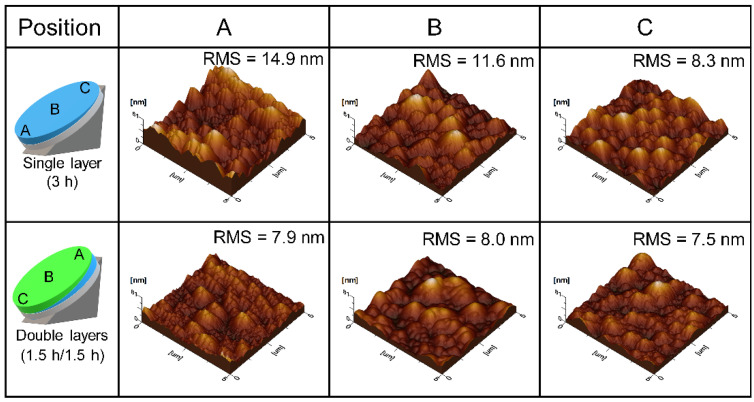
Roughness of the single- and double-layer samples at different positions (**A**–**C**).

Figure 3 shows the AFM images of the single and double Ga_2_O_3_ layers at different substrate positions (A, B, and C). Figure 3 shows the three-dimensional AFM images of the layers with an area of 5 µm × 5 µm. In addition to the change in the layer thickness, the single Ga_2_O_3_ layer exhibited a significantly varying surface roughness. The root mean square (RMS) surface roughness of positions A, B, and C were 14.9, 11.6, and 8.3 nm for the single Ga_2_O_3_ layer, and 7.9, 8.0, and 7.5 nm for the double Ga_2_O_3_ layer, respectively. The standard deviation of the RMS surface roughness of the single Ga_2_O_3_ layer was as large as 3.3 nm, which is predictable for a single epitaxial layer prepared by the horizontal mist CVD [24]. However, the standard deviation of the RMS surface roughness was 0.3 nm for the double Ga_2_O_3_ layer, indicating the uniform surface roughness of the double Ga_2_O_3_ layers. Hence, the two-step growth significantly improves the roughness uniformity of the final Ga_2_O_3_ epitaxial layer.

**Figure 4:**
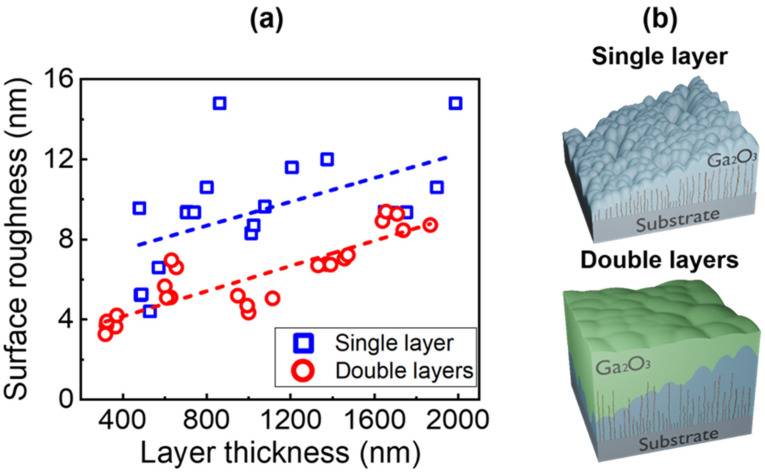
(**a**) Dependence of the surface roughness on the epitaxial layer thickness. (**b**) Schematic of the structural growth behaviors of the single layer and double layers.

Figure 4a shows the plot of the RMS surface roughness of the Ga_2_O_3_ layers with a change in the thickness. At first glance, the overall RMS surface roughness of the Sn-doped Ga_2_O_3_ layers fabricated in this study were several times higher than that of unintentionally-doped Ga_2_O_3_ layers reported in a previous study (0.8–3.7 nm) [24]. It was explained by the segregation of Sn dopants during growth, which increases significantly under a Ga-rich VI/III ratio [26]. Moreover, the RMS values of both the single and double Ga_2_O_3_ layers increased with the thickness of the grown layer. The double Ga_2_O_3_ layers exhibited a more notable improved smoothness compared to the single Ga_2_O_3_ layer. These results indicate that the two-step growth with a substrate rotation of 180° was effective for improving the uniformity of the substrate and surface morphology (Figure 4b).

### 3.2. Crystal Quality

**Figure 5:**
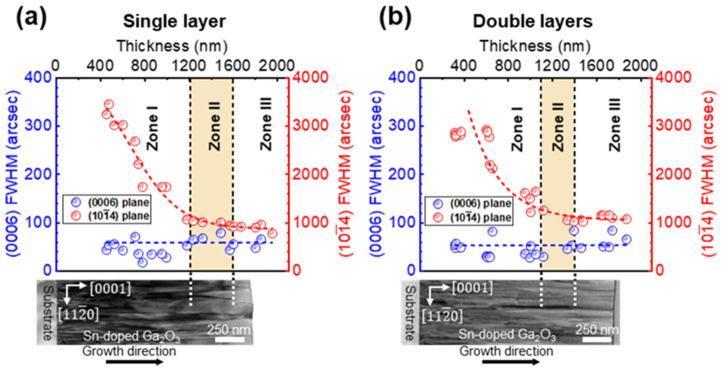
Full width at half maximum values of the two layers as a function of the Ga_2_O_3_ epitaxial thickness of the: (**a**) single-layer and (**b**) double-layer samples. The transmission electron microscopy (TEM) images show the propagation of edge dislocation associated with the FWHM.

Figure 5 shows the full width at half maximum (FWHM) plots of the ω-scan XRD rocking curve of the symmetrical (0006) and asymmetrical (10$\bar{1}$4) planes as a function of the Ga_2_O_3_ layer thickness. Both the single and double Ga_2_O_3_ layers maintained relatively low FWHMs values for the symmetrical (0006) plane reflection over the entire thickness, which ranged from 40–90 arcsec, indicating the highly preferred crystal orientation of the layers along the surface normal direction (i.e., growth direction). In contrast, the FWHM values of the asymmetric (10$\bar{1}$4) plane reflections were relatively higher than those of the (0006) plane. As the thickness of the layers increased, the FWHMs of the (10$\bar{1}$4) plane reflections rapidly decreased and was saturated to ≈1000 arcsec at a certain thickness (e.g., ≈1.5 µm). The large discrepancy in the FWHM values of the (0006) and $\left(10\bar{1}4\right)$ planes are commonly attributed to the edge and screw dislocations density, respectively, wherein the edge dislocation density is relatively higher than the screw dislocation density [27]. This indicates that the improved crystallinity of the epitaxial layers could be attributed to the reduction in the edge dislocation density with an increase in the thickness until saturation. The reduction in the edge dislocation was further confirmed by the TEM analysis of the area along the 〈$10\bar{1}0$〉 zone axis in the upper panel of Figure 5a,b. To observe the full variation over the growth thickness, ≈1700-nm-grown Ga_2_O_3_ layers of both the single and double Ga_2_O_3_ layers were selected. Owing to the growth of homoepitaxial layers in the double Ga_2_O_3_ layers, the interface between the two layers in the double Ga_2_O_3_ layers was not observed. In addition, dense dark lines were observed at the interface of the Ga_2_O_3_ layer and sapphire substrates, indicating the occurrence of a high density of threading dislocations at the initial growth stage. As the growth proceeded, the dislocation density of the single and double Ga_2_O_3_ layers decreased. This indicates that the reduction in the edge dislocations resulted in a high crystal quality of the Ga_2_O_3_ layers with an increase in the growth thickness. In summary, the improvement in the crystal quality on the FWHM $\left(10\bar{1}4\right)$ plane XRD rocking curve was similarly saturated at certain thicknesses by thickening both the single and double Ga_2_O_3_ layers (≈1.2 and ≈1.0 µm, respectively). The Hall measurement at room temperature (RT) was conducted for investigating the electrical properties of the single and double layers. For both of them, the low-quality, thin Ga_2_O_3_ layers are unable to measure the Hall’s effect at RT, whereas the high-quality, thick Ga_2_O_3_ layers are able to measure the Hall’s effect. Therefore, the grown Ga_2_O_3_ layers were divided into three zones depending on the crystal quality and Hall measurability at RT as follows:

- Zone I: Thin layer (<1.1 µm), Hall effect unmeasurable at RT, low crystal quality.
- Zone II: Transition layer, Hall effect unmeasurable, saturated crystal quality.
- Zone III: Thick layer (>1.6 µm), Hall effect measurable at RT, high crystal quality.

The largest discrepancy between the single and double Ga_2_O_3_ layers was observed at the transition layer (Zone II). The thickness of the transition layer of the single Ga_2_O_3_ layer was in the range from 1.2–1.6 µm. Despite the saturation of the enhancement in the crystal quality, the single, one-step growth required a further elongation of the high-quality region. In contrast, the thickness of the transition layer of the double Ga_2_O_3_ layers ranged from 1.1–1.4 µm. Compared to the single Ga_2_O_3_ layer, the thickness of the double Ga_2_O_3_ layer left-shifted and reduced, indicating that the Hall effect was measurable before the double Ga_2_O_3_ layer reached the highest crystal quality. This indicates that a high-quality, conductive Ga_2_O_3_ layer is achievable at a relatively low thickness using the two-step growth procedure, suggesting an advantage of the two-step growth method compared to the one-step growth method. The origin of the measurability discrepancy of both layers are discussed in the next section.

### 3.3. Electrical Properties and Scattering Mechanism

**Figure 6:**
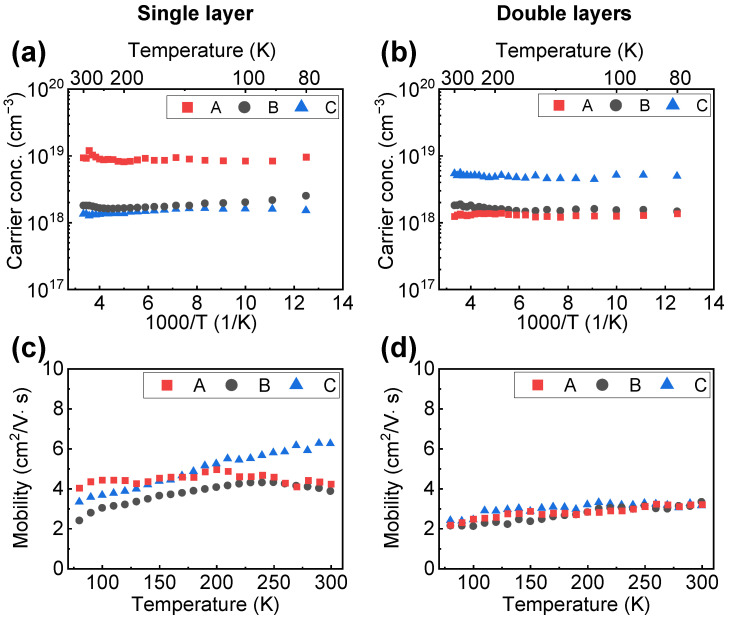
Temperature-dependent carrier concentrations and mobilities of the (**a**,**c**) single layer and (**b**,**d**) double layers of Sn-doped α-Ga_2_O_3_. The average thicknesses of those samples are 1700 nm. A, B, and C are positions located near the wafer flat, center, and edge of the wafer, respectively (see Figure 1).

Figure 6 shows the result of the temperature-dependent Hall measurement of the layers in the range from 80–300 K. The carrier concentrations and mobilities of the two-inch Ga_2_O_3_ epilayers were obtained at three different positions (A, B, and C). A similar thickness of ≈1.7 µm, including a high crystal quality region (Zone III), was selected to minimize the dislocation scattering as the specimen of both the single and double Ga_2_O_3_ layers. The temperature-dependent carrier concentration of the single and double Ga_2_O_3_ layers is shown in Figure 6a,b, respectively. The spatial distribution of the carrier concentration was significantly inhomogeneous depending on the position of the substrate. At a low position in the single Ga_2_O_3_ layer (i.e., point A for the single layer), the carrier concentration (≈1 × 10^19^ cm^−3^) was almost one order higher than those of other positions (≈1 × 10^18^ cm^−3^), as shown in Figure 6a. This high carrier concentration exhibited an almost constant distribution over the entire temperature range owing to the degenerate tendency of free carriers [19]. The same distribution behavior of the temperature-dependent carrier concentration was observed in the double Ga_2_O_3_ layers (Figure 6b). Among the three positions, the lowest position (i.e., position C) of the double Ga_2_O_3_ layers exhibited the highest carrier concentration. In addition, compared to the single layer (≈1 × 10^19^ cm^−3^), the double layer exhibited a slightly lower maximum carrier concentration (≈5 × 10^18^ cm^−3^). The concentration of the other positions (i.e., positions A and B) was almost constant at ≈1 × 10^18^ cm^−3^. It suggests that the inhomogeneous carrier distribution is inevitable in the horizontal mist CVD system with an inclined substrate holder because of inhomogeneous dopant concentration. The mist flow was highly stagnant at the lower position of the substrate [24], creating a higher doping efficiency at that position. It implies that a low mist flow velocity can increase the doping efficiency. Moreover, the spatial gradient of the carrier concentration was strongly affected by Sn concentration distribution at the surface, regardless of the Sn concentration distribution of the underlying layer.

The mobility distribution of the single and double Ga_2_O_3_ layers over the entire temperature range is shown in Figure 6c,d. Regardless of the maximum carrier concentration, position A on the single Ga_2_O_3_ layer exhibited the largest mobility in the low-temperature range of 80–150 K (Figure 6c). According to Matthiessen’s rule, the accumulated mobility µ is defined by the components mobility as described in Equation (1):

1/µ = 1/µ_c_ + 1/µ_p_+ 1/µ_s_ (1)

where T, µ_c_, µ_p_, and µ_s_ are temperature, Coulomb scattering mobility, phonon scattering mobility, and surface roughness scattering, respectively [19,20]. The dislocation scattering was excluded in Zone III, where the dislocation was small and crystal quality was high (See also the schematic descriptions of the mobility variation depending on scattering effects in Figure S1a, Supplementary Materials) [22,28]. Therefore, high mobility at position A could be attributed to the higher effective thickness and high crystal quality at that position compared to other positions, which were susceptible to low dislocation scattering. Moreover, Coulomb scattering at low temperatures and phonon scattering at high temperatures are proportionally related to T^3/2^ and T^−3/2^. Hence, the maximum point in position B of the single layer over the whole temperature was observed at 220 K, then the mobility decreases due to phonon scattering. Owing to the maximum carrier concentration of position A on the single Ga_2_O_3_ layer, the maximum point shifted to a lower temperature of 200 K and the mobility variation was smaller than that of position B. In contrast, the mobility peak of position C shifted to the higher temperature (300 K) with the highest value of 6.5 cm^2^/V∙s. As position C of the single Ga_2_O_3_ layer exhibited the lowest carrier concentration, the peak shifting behavior to higher temperatures can be considered reasonable. Furthermore, the smooth surface roughness of position C (RMS surface roughness ≈8.3 nm) could result in a sizeable difference from that of position B (RMS surface roughness ≈11.6 nm) on the single Ga_2_O_3_ layer, suggesting that the surface roughness scattering considerably influences the carrier mobility in the single Ga_2_O_3_ layer [22,29].

In contrast, the carrier mobility of the double Ga_2_O_3_ layers slightly and monotonically increased as the temperature increased (Figure 6d). The carrier mobility gradually increased from ≈2 cm^2^/V∙s and peaked at ≈2.5 cm^2^/V∙s, which was similarly obtained at 300 K for all three positions. With the layer thickness of 1.7 μm, the crystal quality and surface roughness of the double Ga_2_O_3_ layers were good and uniform on all three positions. Therefore, the dislocation and surface roughness scattering could not affect the temperature-dependent characteristics of carrier mobility in the double Ga_2_O_3_ layer. On the other hand, the double Ga_2_O_3_ layers were susceptible to strong Coulomb scattering owing to the heavily n-doped Ga_2_O_3_ (1 × 10^19^ cm^−3^) and, consequently, just slightly increased the mobility with temperature. Moreover, the double layers seem to be less affected by phonon scattering and are promising for working at elevated temperatures. Hence, achieving a high electron mobility with lightly doped n-Ga_2_O_3_ layers is essential for the Ga_2_O_3_-based device applications.

## 4. Conclusions

In summary, Sn-doped Ga_2_O_3_ epitaxial layers were grown on a two-inch sapphire substrate using horizontal mist CVD. The microstructural gradational properties, such as thickness, surface morphology, and dislocation density, of single- and double-grown Ga_2_O_3_ layers were compared. The single Ga_2_O_3_ layer exhibited a considerable microstructural gradation, which could be attributed to the spatial difference in the flow of mist to the substrates. The bottom of the single Ga_2_O_3_ layer exhibited a higher thickness and surface roughness, whereas the upper positions of the substrates exhibited gradually reduced thickness and surface roughness. The microstructural gradational properties of the single Ga_2_O_3_ layer resulted in significant deviation in the electrical properties of the layer in terms of carrier concentration and mobility. In contrast, the double Ga_2_O_3_ layers exhibited excellent thickness uniformity, surface roughness, and crystal quality compared to the single Ga_2_O_3_ layer. Nevertheless, the electron mobility of the double Ga_2_O_3_ layers was ≈2.5 cm^2^/Vs with small variations over a wide range of temperature. These results indicate that the carrier mobility of Ga_2_O_3_ layers can be improved with lightly-doped n-Ga_2_O_3_ layers. Therefore, the two-step mist CVD growth can prepare a high and homogeneous quality Ga_2_O_3_ epitaxial layer for high-temperature and high-power applications.

## Data Availability

Data is contained within the article.

## Supplementary Materials

The following supporting information can be downloaded at: https://www.mdpi.com/article/10.3390/ma15031050/s1, Figure S1: Schematic description of the temperature-dependent carrier mobility of the grown layers under the effects of various scattering mechanisms. Figure S2: Omega-scan XRD patterns and FWHM of (0006) and (10$\bar{1}$4) planes for single and double layers with different thicknesses. Table S1: Hall measurement data of the temperature-dependent carrier mobility and carrier concentration of single and double layers.
