# Supplementary material for: Microstructural Gradational Properties of Sn-Doped Gallium Oxide Heteroepitaxial Layers Grown Using Mist Chemical Vapor Deposition
Source: Materials (Basel). 2022 Jan 29;15(3):1050. doi: 10.3390/ma15031050 (PMC8838345; doi:10.3390/ma15031050)
Supplement: Supplementary file 1 [file materials-15-01050-s001.zip › materials-1547891-SI.pdf]

Supporting Information

Microstructural Gradational Properties of Sn-Doped Gallium Oxide Heteroepitaxial Layers Grown Using Mist Chemical Vapor Deposition

Kyoung-Ho Kim [1,2], Minh-Tan Ha [3], Heesoo Lee [2], Minho Kim [4], Okhyun Nam [4], Yun-Ji Shin [1], Seong-Min Jeong [1] and Si-Young Bae [1,*]

[1] Semiconductor Materials Center, Korea Institute of Ceramic Engineering and Technology, Jinju 52851, Korea; energykkh@gmail.com (K.-H.K.); shinyj@kicet.re.kr (Y.-J.S.); smjeong@kicet.re.kr (S.-M.J.)
[2] School of Materials Science and Engineering, Pusan National University, Busan 46241, Korea; heesoo@pusan.ac.kr
[3] School of Materials Science and Engineering, Changwon National University, Changwon 51140, Korea; haminhtan.mse@gmail.com
[4] Convergence Center for Advanced Nano Semiconductor (CANS), Department of Nano & Semiconductor Engineering, Korea Polytechnic University, Siheung-si, Gyeonggi-do 15073, Korea; First_kim88@naver.com (M.K.); ohnam@kpu.ac.kr (O.N.)
* Correspondence: sybae@kicet.re.kr

Citation: Kim, K.-H.; Ha, M.-T.; Lee, H.; Kim, M.; Nam, O.; Shin, Y.-J.; Jeong, S.-M.; Bae, S.-Y. Microstructural Gradational Properties of Sn-Doped Gallium Oxide Heteroepitaxial Layers Grown Using Mist Chemical Vapor Deposition. *Materials* **2022**, *15*, 1050. https://doi.org/10.3390/ma15031050

Academic Editor: Lei Zhang

Received: 24 December 2021
Accepted: 26 January 2022
Published: 29 January 2022

Publisher's Note: MDPI stays neutral with regard to jurisdictional claims in published maps and institutional affiliations.

Copyright: © 2022 by the authors. Submitted for possible open access publication under the terms and conditions of the Creative Commons Attribution (CC BY) license (https://creativecommons.org/licenses/by/4.0/).

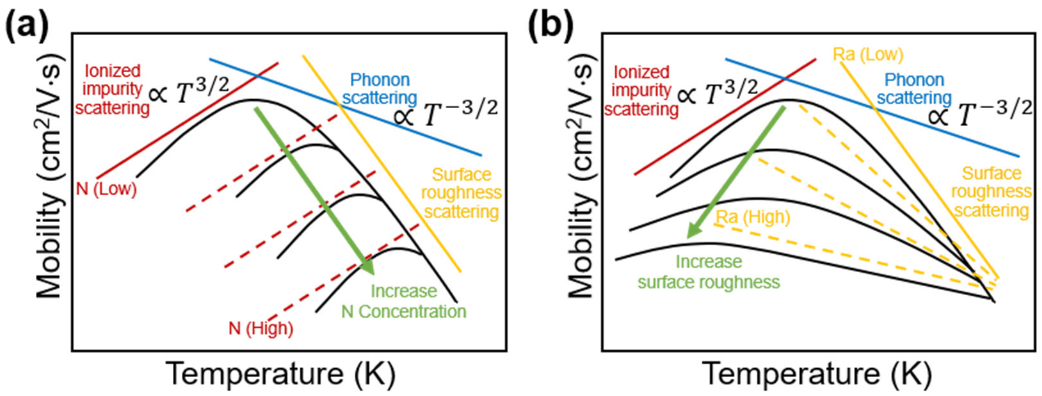

Figure S1. Schematic description of the temperature-dependent carrier mobility of the grown layers under effects of various scattering mechanisms: (a) variation in impurity concentration and (b) variation in surface roughness. Note that the peak shifts to high temperature and peak shifts to low temperature with increasing N concentration and surface roughness, respectively [1,2].

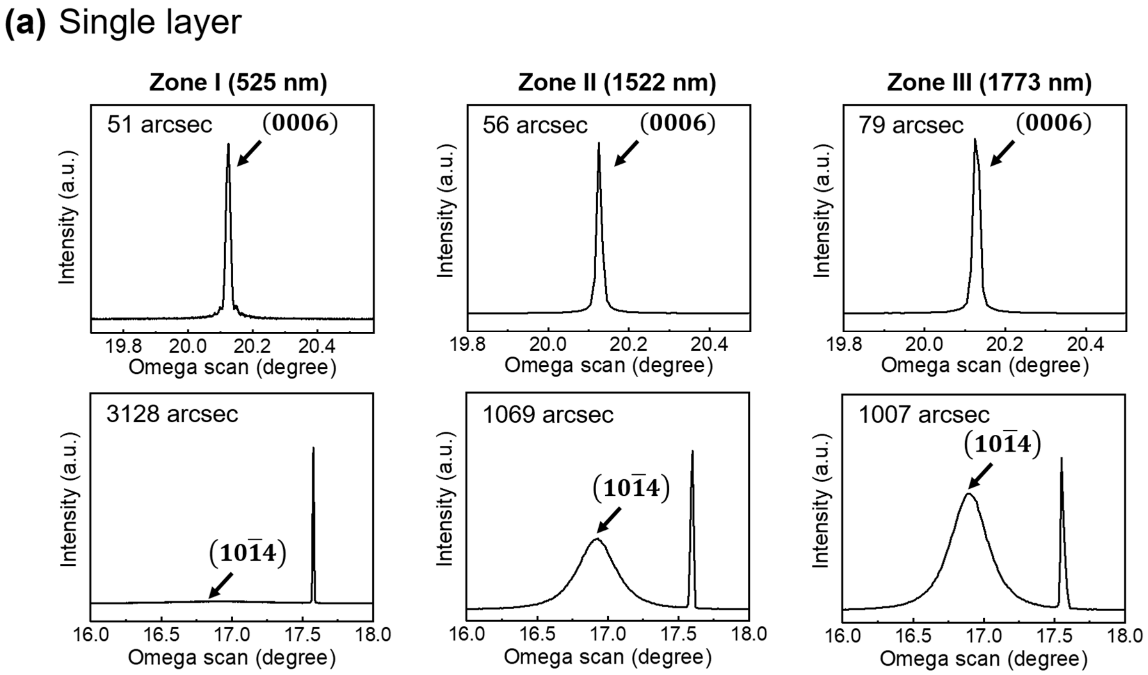

(b) Double layers

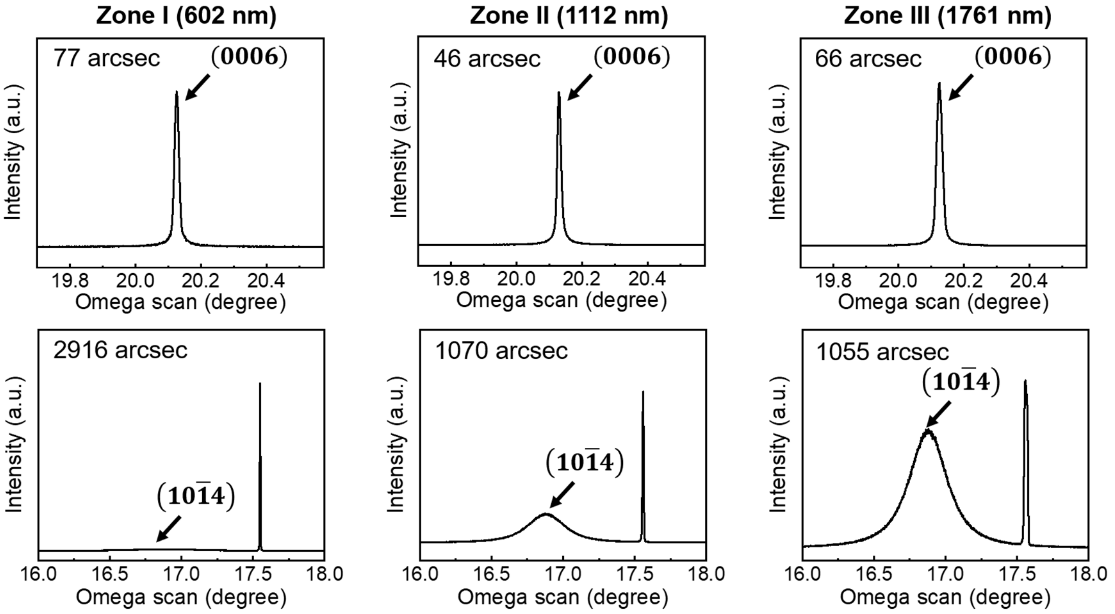

Figure S2. (0006) and (10$\bar{1}$4) plane omega-scan XRD patterns and FWHM for (a) single Ga_2O_3 layer and (b) double Ga_2O_3 layers with various thicknesses.

Table S1. Hall measurement data of the temperature-dependent carrier mobility and carrier concentration for (a) single Ga_2O_3 layer and (b) double Ga_2O_3 layers.

	**Single layer**					
	mobility (cm^2/V·s)			carrier concentration (×10^{18} cm^{-3})		
**Temp. (K)**	A	B	C	A	B	C
80	4.0	2.4	3.3	1.3	1.8	9.4
90	4.4	2.8	3.6	1.3	1.8	9.2
100	4.4	3.0	3.7	1.3	1.8	12.0
110	4.4	3.1	3.8	1.3	1.7	10.3
120	4.4	3.2	3.9	1.3	1.7	9.6
130	4.3	3.3	4.0	1.3	1.7	9.0
140	4.4	3.5	4.2	1.4	1.6	8.8
150	4.5	3.6	4.4	1.3	1.6	8.9
160	4.6	3.7	4.4	1.4	1.6	8.8
170	4.6	3.8	4.6	1.4	1.6	8.3
180	4.6	3.9	4.9	1.4	1.6	8.1
190	4.8	4.0	5.1	1.4	1.7	8.3
200	5.0	4.1	5.2	1.4	1.7	8.7
210	4.9	4.1	5.5	1.5	1.7	9.2
220	4.6	4.2	5.4	1.5	1.7	8.6
230	4.6	4.3	5.5	1.5	1.7	8.6
240	4.7	4.3	5.7	1.6	1.8	9.5
250	4.6	4.3	5.8	1.6	1.8	9.0
260	4.3	4.2	5.8	1.6	1.9	8.6
270	4.1	4.1	6.2	1.6	2.0	8.5
280	4.4	4.1	5.9	1.6	2.0	8.3
290	4.3	4.0	6.3	1.6	2.2	8.4
300.	4.2	3.9	6.3	1.5	2.5	9.6

(b)

	Double layers					
	mobility (cm^2/V·s)			carrier concentration (×10^{18} cm^{-3})		
Temp. (K)	A	B	C	A	B	C
80	2.4	2.1	2.2	5.4	1.8	1.2
90	2.4	2.1	2.3	5.1	1.8	1.3
100	2.4	2.1	2.5	5.5	1.9	1.4
110	2.9	2.3	2.5	5.0	1.7	1.3
120	2.9	2.3	2.6	5.1	1.7	1.3
130	2.9	2.2	2.8	5.0	1.8	1.3
140	3.0	2.4	2.8	5.1	1.6	1.3
150	2.9	2.3	2.9	5.0	1.7	1.4
160	3.0	2.5	2.7	4.8	1.7	1.4
170	3.1	2.6	2.8	4.7	1.6	1.4
180	3.1	2.7	2.8	4.8	1.6	1.3
190	3.0	2.7	2.7	5.1	1.6	1.4
200	3.2	2.8	2.8	4.8	1.6	1.3
210	3.3	3.0	2.8	4.7	1.5	1.3
220	3.2	3.0	2.9	4.6	1.5	1.3
230	3.2	3.1	2.9	5.0	1.5	1.2
240	3.2	3.0	3.0	4.6	1.6	1.2
250	3.3	3.1	3.1	4.6	1.5	1.2
260	3.2	3.0	3.2	4.6	1.6	1.3
270	3.2	3.0	3.2	4.4	1.6	1.3
280	3.0	3.1	3.1	5.1	1.5	1.3
290	3.2	3.1	3.2	5.1	1.6	1.3
300	3.1	3.3	3.2	5.0	1.5	1.4

References

1. Shin, H.; Lim, K.; Hwang, S.; Han, I.-K.; Jang, M. The Evaluation of Hole Mobility Characteristics with Surface Roughness. *Journal of Nanoscience and Nanotechnology* **2017**, 17, 7766–7770, doi:10.1166/jnn.2017.14840.
2. Noguchi, M.; Iwamatsu, T.; Amishiro, H.; Watanabe, H.; Kita, K.; Yamakawa, S. Determination of Intrinsic Phonon-Limited Mobility and Carrier Transport Property Extraction of 4H-SiC MOSFETs. In Proceedings of the 2017 IEEE International Electron Devices Meeting (IEDM); IEEE: San Francisco, CA, USA, December **2017**; p. 9.3.1-9.3.4.
